# Complete Genome Sequence of *Spiroplasma cantharicola* CC-1^T^ (DSM 21588), a Bacterium Isolated from Soldier Beetle (*Cantharis carolinus*)

**DOI:** 10.1128/genomeA.01253-15

**Published:** 2015-10-22

**Authors:** Wen-Sui Lo, Po-Yu Liu, Chih-Horng Kuo

**Affiliations:** a Institute of Plant and Microbial Biology, Academia Sinica, Taipei, Taiwan; b Molecular and Biological Agricultural Sciences Program, Taiwan International Graduate Program, National Chung Hsing University and Academia Sinica, Taipei, Taiwan; c Graduate Institute of Biotechnology, National Chung Hsing University, Taichung, Taiwan; d Genome and Systems Biology Degree Program, National Taiwan University and Academia Sinica, Taipei, Taiwan; e Department of Life Science, National Taiwan University, Taipei, Taiwan; f Biotechnology Center, National Chung Hsing University, Taichung, Taiwan

## Abstract

*Spiroplasma cantharicola* CC-1^T^ (DSM 21588) was isolated from the gut of a soldier beetle (*Cantharis carolinus*) collected in Maryland, USA. Here, we report the complete genome sequence of this bacterium to facilitate the investigation of its biology.

## GENOME ANNOUNCEMENT

*Spiroplasma cantharicola* is an insect-associated bacterium found in Maryland, USA (1). This species belongs to the XVI-1 subgroup within the genus *Spiroplasma*, which includes strains isolated from beetle (*Cantharis carolinus*; represented by strain CC-1^T^) and wasp (*Monobia quadridens*; represented by strain MQ-6) (2). Other serologically related strains with less than 70% similarities in DNA-DNA hybridization tests have been assigned to subgroups XVI-2 and XVI-3 (2). Of these, XVI-2 includes strains isolated from beetle (*Cantharis bilineatus*) and mosquito (*Aedes fulvus/annulipes* and *Anopheles punctipennis*) collected in the United States. In contrast, XVI-3 strains were all isolated from the Savoy region of France. Most of the XVI-3 strains are associated with mosquito (*Aedes* spp. and *Coquillettidia richiardii*; represented by strain Ar-1357). The XVI-3 strain PI-30L was isolated from a thistle plant (*Cirsium* sp.), representing a notable exception in terms of host association. To facilitate future investigation into the biology of these bacteria, as well as to improve the taxon sampling of available *Spiroplasma* sequences for comparative genomics and evolutionary studies, we determined the complete genome sequence of *S. cantharicola* CC-1^T^.

The procedures for sample processing, sequencing, assembly, and annotation were based on those described in our previous studies on *Spiroplasma* genomes (3–9). The strain was acquired from the German Collection of Microorganisms and Cell Cultures (catalogue no. DSM 21588). The freeze-dried sample was processed according to the manufacturer’s instructions and cultured in the M1D medium (10) prior to DNA extraction. PCR and Sanger sequencing were performed to verify that the 16S rRNA gene sequence matched the reference record (GenBank accession no. NR_125516.1).

The Illumina MiSeq platform was used to generate 301-bp reads from one paired-end library (~580-bp insert; 1,323,240,548 reads). The initial *de novo* assembly was performed using Velvet version 1.2.10 (11). Subsequently, PAGIT version 1 (12) was used to assist an iterative process for improving the assembly. For each iteration, the raw reads were mapped to the assembly using BWA version 0.7.12 (13), programmatically checked using the MPILEUP program in the SAMTOOLS package version 1.2 (14), and visually inspected using IGV version 2.3.57 (15). Polymorphic sites and gaps were corrected based on the mapped reads. The process was repeated until the complete genome sequence was obtained.

The programs RNAmmer (16), tRNAscan-SE (17), and Prodigal (18) were used for gene prediction. The gene names and product descriptions were first annotated based on the homologous genes in other *Spiroplasma* genomes (3–9) as identified by OrthoMCL (19). Subsequent manual curation was based on BLASTp (20) searches against the NCBI nonredundant database (21) and the KEGG database (22, 23).

The circular chromosome of *S. cantharicola* CC-1^T^ is 1,179,577 bp in size and has a G+C content of 25.0%; no plasmid was found. The first version of annotation includes one set of 16S-23S-5S rRNA genes, 29 tRNA genes (covering all 20 amino acids), and 1,017 protein-coding genes.

### Nucleotide sequence accession number.

The complete genome sequence of *S. cantharicola* CC-1^T^ has been deposited in DDBJ/EMBL/GenBank under the accession number CP01262.
